# Has Smartphone Use Influenced Loneliness during the COVID-19 Pandemic in Japan?

**DOI:** 10.3390/ijerph191710540

**Published:** 2022-08-24

**Authors:** Trinh Xuan Thi Nguyen, Sumeet Lal, Sulemana Abdul-Salam, Pattaphol Yuktadatta, Louis McKinnon, Mostafa Saidur Rahim Khan, Yoshihiko Kadoya

**Affiliations:** 1 School of Economics, Hiroshima University, 1-2-1 Kagamiyama, Higashihiroshima 7398525, Japan; 2 Department of Economics, University of California, Berkeley, 530 Evans Hall # 3880, Berkeley, CA 94720-3880, USA

**Keywords:** loneliness, smartphone, COVID-19 pandemic, Japan

## Abstract

The influence of smartphone use on increased risk of feeling lonely has been recognized as a global public health concern. However, it is unclear whether this influence has changed during the ongoing COVID-19 pandemic, during which smartphones have become a particularly important means of communication due to health safety measures restricting personal interactions. We used Hiroshima University’s online survey data collected from 18–28 February 2022, to assess the impact of smartphone use on loneliness in Japan. The final sample included 2630 participants aged over 20 years, with loneliness measured using the UCLA scale and smartphone use calculated as the duration of usage in minutes/day. Weighted logit regression analysis was used to examine the association between smartphone use and loneliness, with other demographic, socioeconomic, and psychological characteristics as explanatory variables. Contrary to conventional evidence, our findings show that smartphone use mitigated the risk of loneliness during the pandemic. This was especially true among females under 65 years old. We found that age, subjective health status, future anxiety, and depression impacted this relationship. The findings of this study can help guide policymaking by showing the importance of providing adequate digital platforms to manage loneliness and mental health during times of isolation.

## 1. Introduction

The influence of smartphone use on increased risk of loneliness has become a public health concern worldwide [1,2]. Researchers have provided substantial evidence that prolonged smartphone use increases the likelihood of loneliness. For example, a meta-analysis of 134 independent studies showed that loneliness and smartphone addiction are positively correlated [3]. Other research also suggests that extended smartphone use exacerbates feelings of loneliness [4] and leads to poorer mental health outcomes [5,6]. However, whether this relationship remains the same in unexpected circumstances or disaster situations, such as the COVID-19 pandemic, is understudied and of great interest to academics and the general public.

This study examined the association between loneliness and smartphone use in Japan during the ongoing COVID-19 pandemic using data collected in 2022. Despite the positive correlation between loneliness and smartphone use under normal conditions, smartphones have played an essential role in helping people stay socially connected with others amid strict lockdown measures [7]. Although unable to fully compensate for real-life physical contact, technology-based solutions have high potential for maintaining social relations [8]. Particularly among young people, smartphones allow people to keep in touch with their loved ones from the confines of their homes [9,10].

Moreover, the importance of exploring the association between smartphone use and loneliness during a pandemic has been highlighted by previous studies. On the one hand, prior research has provided important implications that smartphone use anticipated higher levels of loneliness during the outbreak of COVID-19 [11,12,13]. This is supported by the previous findings that digital technology utilization amplified public distress during disasters other than the COVID-19 pandemic. For example, during the Hong Kong tsunami in late December 2004, Lau et al. [14] reported the intensifying effect of mass media use on people’s mental health, and the same association was found after the 2013 Boston Marathon bombings [15]. On the other hand, social media can be adopted as a constructive method to cope with negative feelings experienced during COVID-19 quarantines [16], suggesting that the use of smartphones may be linked to lower levels of loneliness in young adults [17].

In fact, smartphone use has been shown to have a bidirectional effect on loneliness, which can be explained by two underlying hypotheses [18]. The “stimulation hypothesis” postulates that if internet use via portable technology is utilized to facilitate offline relationships or to build new friendship [19,20], users receive more social support, thus being less likely to feel lonely. The “displacement hypothesis,” on the other hand, suggests that when social technologies are used in a way that displaces offline interactions with online interactions and/or activities, they are associated with increased loneliness [21,22,23]. In contrast, when social technologies are used to forge new friendships and enhance existing ones, social internet use can lead to reductions in loneliness, but only when there is an overlapping of the offline and online social networks [21,22,23]. Thus, the level of loneliness depends on how displacement contributes to social interactions as well as the maintenance of relationship. If the level of engagement of an individual with peers is higher in an online relationship compared to an offline relationship, the association with loneliness depends on how they manage their offline ones. Therefore, given the complex nature and contrasting findings of previous studies, more research on the association between loneliness and smartphone use may have significant implications for public health efforts during the COVID-19 pandemic.

To the best of our knowledge, this study is the first to explore empirical evidence of the relationship between smartphone use and loneliness after the onset of the pandemic in Japan. Most articles on loneliness in Japan have mainly addressed its prevalence [24,25] and its association with other behavioral and psychological symptoms [26,27,28]. Therefore, together with the need to understand the correlation between smartphone use and loneliness, the primary purpose of this study was to further investigate this association under the effects of the COVID-19 pandemic in Japan. Furthermore, since loneliness varies by age and gender [29], our secondary focus was to investigate the role of smartphone use across specific age groups and sexes within the Japanese population. Our study contributes to the existing body of literature in at least two ways. First, we report evidence of the relationship between smartphone use and loneliness during the pandemic in Japan across different age and gender strata. Second, we provide a clearer picture of the potential effects of smartphone use on loneliness and mental health. Thus, this study identifies vital information for policymakers in Japan and other countries to understand the role of smartphone in loneliness and mental health during a pandemic and to develop relevant policy accordingly.

## 2. Data and Methods

### 2.1. Data

The data used in this is a subset of data of the Household Behavioral and Financial Survey organized by Hiroshima University. The panel dataset was collected online by Nikkei Research, which is one of the top research companies in Japan. Questionnaires were distributed to each prospective participant, and the number of participants was measured using a random sampling procedure to ensure the representativeness of the panel data. The survey included questions on preferences and demographic, socioeconomic, and psychological aspects of Japanese adults with a minimum age of 20 years and was conducted over three years of the COVID-19 pandemic (2020, 2021, and 2022). The first data collection occurred from 20–25 February 2020, at the beginning of the pandemic, with a total of 17,463 participants, and 6103 of the original sample chose to participate in the second wave of the survey from 19–26 February 2021. The third round of data collection was between 18–28 February 2022, with a sample size of 4281. In this study, we used several demographic variables from the 2020 wave, while other variables of interest were from the 2022 wave. Thus, the final merged dataset in two years had 2630 observations after removing some missing socioeconomic values of financial literacy, household income, household assets, and employment status.

### 2.2. Variables

In this study, the dependent variable was “loneliness,” which was a binary variable measuring loneliness in 2022. We adopted the UCLA methodology [30] to measure loneliness through three questions, namely “How often do you feel a lack of companionship”, “How often do you feel left out”, and “How often do you feel isolated from others”? Answer options were “Hardly ever or never”, “Some of the time”, and “Often”. Participants were classified as lonely (loneliness = 1) if they responded that they felt a lack of companionship, left out, and isolated at least some of the time, and 0 otherwise.

“Smartphone use”, the main independent variable of our study, was measured in minutes per day via the question “On average, how many hours do you use your smartphone per day”? In addition, we obtained static demographic variables, such as gender, child-rearing status, residence, and years of education, from the 2020 dataset. The 2020 financial literacy variable was also included, representing rational financial and health behaviors [31,32,33]. Other socioeconomic variables from the 2022 wave were utilized, including age, marital status, living status, employment status, and household financial status. The study also included subjective health status, depression, and other perception variables, such as future anxiety, financial satisfaction, and a myopic view of the future. Most of our explanatory variables were comparable to those in the two articles of Khan and Kadoya [29] and Khan et al. [34]. Table 1 provides a clear definition for each variable.

### 2.3. Descriptive Statistics

According to the descriptive statistics presented in Table 2, 65% of the respondents in 2022 were lonely based on the UCLA measure. Interestingly, respondents spent an average of 121 min per day using their mobile devices. In terms of demographics, our results revealed that approximately 70% of the participants were male and that the average age of our sample was approximately 53.83, which was almost similar to the median age 54. Furthermore, the surveyed people completed an average of 15 years of education, and 63.9% worked full time. The rural population accounted for 57.3% of participants, and the average financial literacy score was 0.70. Considering the composition of households in 2022, 67% had a spouse or lived with a partner, 20% lived on their own, and 58% had a child or children. Regarding financial status, the average participant lived in a household with a total income of 6.50 million yen per year and total assets of 24.1 million yen. Finally, the results showed that during the 2022 pandemic, respondents rated scores for subjective health status, future anxiety, financial satisfaction, depression, and future orientation at 3.27, 3.78, 2.85, 2.89 and 2.68 out of 5, respectively.

The entire sample was divided into subsamples stratified according to sex and age. Table 3 indicates that a mean difference at the 99% significance level was seen in loneliness across the younger and older groups for both sexes. Specifically, the percentage of young people who were lonely in 2022 was consistently higher than that of their older counterparts regardless of gender.

### 2.4. Methods

We analyzed the association between loneliness and demographic, socioeconomic, psychological, and health-related factors using Equation (1):

*Y*1*i* = f(*Xi*, *εi*), (1)

where *Y*1*i* is loneliness of the *i*th respondent in 2022; *X* is a vector of individuals’ demographic, socioeconomic, psychological, and health-related characteristics; and *ε* is the error term. As our dependent variable was binary, we performed a cross-sectional weighted logit regression to estimate the equation.

Correlation and multicollinearity tests were used to identify and compensate for the high intercorrelations between two or more independent variables (results available upon request). The correlation matrix revealed a weak association between the relative movements of the two variables (substantially lower than 0.70). Furthermore, all the explanatory variables’ variance inflation factor were less than five, indicating that multicollinearity was not present in any of the models. In addition to the multicollinearity problem, we used weighted regression to standardize the sample population to the total population to improve the representativeness of our study. In particular, we estimated sampling weights by dividing the total population collected from the 2020 Population Census [35] by sampling populations stratified by age and gender. The distribution of male and female across different age groups in the 2020 Population Census is shown in Appendix A.

Based on the models adopted in the studies conducted by Khan and Kadoya [29] and Khan et al. [34], the full specification for Equation (1) in our study is represented in the model below:

$$\begin{array}{c} \ln\left[\frac{P\left(Yi\right)}{1-P\left(Yi\;\right)}\right]=\beta 0+\beta 1smart\;phone\;usei+\beta 2malei+\beta 3agei+\beta 4spousei+\beta 5childreni+\beta 6living\;alonei+\beta 7living\;in\\ \;rural\;areasi+\beta 8educationi+\beta 9full-time\;employmenti+\beta 10logofhousehold\;incomei+\beta 11logof\;household\;assetsi+\\ \beta 12financial\;literacyi+\beta 13subjective\;healthi+\beta 14future\;anxietyi+\beta 15financial\;satisfactioni+\beta 16depressioni+\\ \beta 17myopic\;view\;of\;the\;future \end{array}$$

where $\ln\left[\frac{P\left(Yi\right)}{1-P\left(Yi\;\right)}\right]$ is the log(odds) of the outcomes. *Yi* = 1 if the *i*th respondent felt lonely in 2022 and *Yi* = 0 if the *i*th respondent did not feel lonely in 2022.

## 3. Results

Table 4 shows the full-sample regression results for loneliness in 2022. In this study, the add-drop method of independent variables was used to test the robustness of our findings, with Model 4 showing the final results for all explanatory variables. In general, most estimates were consistent with our specifications. It is notable that smartphone usage had a negative association with loneliness, which means that the more time people spent interacting with smartphones, the less likely they were to become lonely. Additionally, age, log of household income, and subjective health status were negatively associated with loneliness. In contrast, a positive relationship was observed between future anxiety, depression, and loneliness, implying that people with higher degrees of anxiety about the future or depression tended to suffer from loneliness.

To better understand the changing effects of gender and age group on the association between loneliness and socioeconomic factors, we conducted a subsample analysis between males and their female counterparts, stratified by age group, as shown in Table 5. It should be noted that a negative association between smartphone use and loneliness was only observed among females below 65 years of age. A similar association was observed between subjective health status and loneliness among young females. On the contrary, a higher myopic view of the future was only associated with greater loneliness among young males, while living alone considerably increased the risk of loneliness among older females. Moreover, we discovered that the results for future anxiety and depression were relatively consistent across the four sub-samples; in particular, while older people and young females who were more anxious about the future were more likely to be lonely, an increased risk was witnessed among young people and the older males who experienced higher levels of depression. Significant results were observed for several other variables, including age, educational attainment, household assets, and financial literacy, but they were inconsistent.

## 4. Discussion

Our study revealed that smartphone use was associated with a lower likelihood of loneliness during the pandemic, which contradicts previous research both pre- and during the pandemic. First, our main finding poses a contradictory relationship with the pre-pandemic established notion that increased smartphone use contributes to higher levels of loneliness [36,37]. This is because the data utilized in our study were collected during a special circumstance, the COVID-19 pandemic, when real-life social interactions were limited. In other words, this study revealed the predictive power of social changes as mediating factors in the significant effects of smartphone use on loneliness.

Second, our main result is in contrast to a few earlier studies conducted during the pandemic, which claimed that the use of substitute communication methods, despite becoming extremely popular as a safe replacement for face-to-face conversations, did not always have the same benefits for well-being and mental health as in-person relationships [38,39]. For example, Geirdal et al. [40] suggested that, when compared to less regular social media users, frequent users experienced poorer mental health, a lower quality of life, and felt loneliness. Such studies indicate that while virtual social contacts are preferred over no interactions under the impact of the pandemic, they still appear to lack the benefits that in-person social interactions can provide. Although the data from our study and the articles mentioned above were collected during the same period (i.e., during the ongoing COVID-19 pandemic), one possible explanation for the difference in findings is that how and why smartphones are used is more apposite than the duration of usage in understanding the relationship between usage and loneliness [41].

Finally, our finding of a negative association between smartphone use and loneliness in young Japanese females most closely corresponds with the finding of an earlier Australian study that reported that the use of technology for social connection was linked with lower levels of loneliness in adolescents aged 12–18 [17]. The main difference between these two studies is that we used three measurement questions to give a more rounded picture of loneliness among surveyed respondents, and we also found that the same pattern of a negative association between loneliness and smartphone use did not apply for males or older populations in general. This is probably because older adults usually place higher value on kinship ties, such as societal support and family cohesion [42], and they are less likely to use technological resources than their younger counterparts [8]. Therefore, while social media may have become a constructive approach for young people to confront feelings of loneliness during the COVID-19 pandemic [16], the comprehensive involvement of family, friends, social care service providers, and healthcare professionals would be more practical for older adults. Another probable reason for the significantly negative effect of smartphone use on females is that compared to males, females show a higher tendency to express their feelings and emotions and accumulate more social connections [43]; thus, they are more vulnerable to loneliness due to COVID-19 social isolation and distancing measures.

The main demographic and socioeconomic variables included in this study were age, household income, and household assets. In contrast to several articles that suggested that older populations are most vulnerable to loneliness [44,45], we found no such evidence in our results. More precisely, we noticed that younger people reported significantly higher loneliness than their older counterparts, with a linear decrease with age, which is consistent with the findings of Palgi et al. [46]. This result implies that older adults have more experience with isolation or require fewer social interactions, which ultimately reduces their sensitivity to loneliness.

Household income was unequivocally another strong and robust correlate of general loneliness in 2022. The results showed a negative association between household income and loneliness level, implying that the tendency for an individual to feel lonelier increased as annual household income decreased, which is consistent with recent findings in Japan [47]. In contrast to household income, household assets only had a positive significant relationship with loneliness among older males in the subsample analysis. This result is inconsistent with previous studies reporting that the risk of loneliness decreases as household wealth increases [29,34,48,49]. Alternatively, greater accumulation of wealth indicates a greater responsibility towards maintaining that wealth and sustaining a revenue-generating stream, which may be time-consuming and physically or emotionally draining during a pandemic. Other socioeconomic variables that were found to have no significant relationship with loneliness in 2022 included gender, having a spouse or children, living in rural areas, full-time employment, and financial satisfaction, while education had a minimal and inconsistent association.

The remaining psychological and health concern variables included in our study were anxiety about the future, depression, subjective health status, and a myopic view of the future. Feelings of anxiety about the future and depression were strong predictors of loneliness, with a consistently positive relationship across the subsamples. This is in accordance with the results of a few studies that have shown that depression is associated with subsequent increases in loneliness [29,50,51]. Similarly, for future anxiety, with massive changes in social structures, ranging from job status to caregiving aspects due to the COVID-19 pandemic, people who are more anxious about their future tend to develop social anxiety, which is a perceived threat to interpersonal relations and highly associated with loneliness [52,53,54,55]. Regarding subjective health status, our results showed that people with poorer health conditions tended to suffer from loneliness, which aligns with findings from previous studies [29,56]. Finally, a myopic view of the future was another strong predictor of loneliness, especially among younger males. In addition to having a significant effect on loneliness among older people [29], younger counterparts with a myopic future orientation are also prone to neglect the essence of social connections, resulting in a higher risk of loneliness.

This study had several limitations that need to be considered. First, the number of observations for males was larger and differed considerably from females, and even though weighted regression was employed to reduce the effect, there is a chance that females may have been underrepresented in the sample, possibly creating a bias in the results. Second, our data do not specify the frequency of smartphone use or the reason individuals used their devices, such as browsing news, accessing social media platforms, or for study purposes, all of which might have altered the results in some way. Third, information such as whether a participant was infected with COVID-19 during the time of the survey or if he/she was suffering from any kind of grief is missing from this study, which would likely have had a profound impact on the overall results. Fourth, this was a cross-sectional study, which implies that causal inferences cannot be made; as such, a longitudinal study is needed to establish a causal relationship.

## 5. Conclusions

This study investigated the association between smartphone use and loneliness during the COVID-19 pandemic in Japan. In contrast to widely held beliefs, we hypothesized that increased smartphone use during the pandemic would promote social connection and well-being, ultimately reducing levels of loneliness. Our results were in line with these expectations, and we found that smartphone use was correlated with a reduced risk of developing loneliness, particularly among females who were less than 65 years old. 

The study findings have significant implications for policymakers, government officials, and other researchers. In particular, the results highlight the imperative role that technology, such as smartphones, can play in promoting social connections and acting as a buffer against loneliness, taking into account social contexts. Before the COVID-19 outbreak, smartphones were shown to be a significant factor in increasing loneliness. However, this might not be the case now during the pandemic. To be socially connected amid such quarantine era, people need to be encouraged to use whatever technology is available to them. Although computer-assisted communication technologies are not as effective as face-to-face interactions, they do seem to provide much needed boost towards virtual connectivity and act as a countermeasure against pandemic-derived loneliness.

This was a cross-sectional study, which implies that causal inferences cannot be made and, as such, longitudinal study is needed to provide insights into the aggregated relationship between smartphone use and loneliness, considering their complex interaction in nature. Furthermore, it will be beneficial to determine which specific activities or applications are most effective in fostering social connections.

## Figures and Tables

**Table 1:** Variable definitions.

Variables	Definition
Dependent variable	
Loneliness	Binary variable: 1 = participant reported having feelings of loneliness some of the time or often in 2022, 0 = otherwise
Explanatory variables	
Smartphone use	Continuous variable: number of minutes/day that respondents used their smartphones
Male *	Binary variable: 1 = male, 0 = female
Age *	Continuous variable: respondents’ age in 2022
Spouse	Binary variable: 1 = currently having a spouse or partner, 0 = otherwise
Children *	Binary variable: 1 = having at least one child, 0 = otherwise
Living alone	Binary variable: 1 = living alone, 0 = otherwise
Living in rural areas *	Binary variable: 1 = living in rural areas (not in Tokyo special wards or government-designated city areas), 0 = otherwise
Education *	Discrete variable: years of education
Full-time employment	Binary variable: 1 = having a full-time job, 0 = otherwise
Household income	Continuous variable: annual earned income before taxes and with bonuses of the entire household in 2021 (unit: JPY)
Log of household income	Log (household income)
Household asset	Continuous variable: balance of financial assets (savings, stocks, bonds, insurance, etc.) of the entire household (unit: JPY)
Log of household asset	Log (household asset)
Financial literacy *	Continuous variable: average scores of answers for the three financial literacy questions
Subjective health status	Ordinal variable for the statement, “I am now healthy and was generally healthy in the last one year”. 1 = it does not hold true at all for you, 2 = it is not so true for you, 3 = neither true nor not true, 4 = it is rather true for you, 5 = it is particularly true for you
Future anxiety	Ordinal variable for the statements, “I have anxieties about life after 65 years of age” and “I have anxieties about life in the future” for individuals less than 65 years old and for those who were aged 65 years or above, respectively. 1 = it does not hold true at all for you, 2 = it is not so true for you, 3 = neither true nor not true, 4 = it is rather true for you, 5 = it is particularly true for you
Financial satisfaction	Ordinal variable for the statement, “I am happy with my financial status”. 1 = completely disagree, 2 = disagree, 3 = neither agree nor disagree, 4 = agree, 5 = completely agree
Depression	Ordinal variable for the statement, “I often feel depressed or felt depressed in the last one year”. 1 = it does not hold true at all for you, 2 = it is not so true for you, 3 = neither true nor not true, 4 = it is rather true for you, 5 = it is particularly true for you
Myopic view of the future	Ordinal variable for the statement, “Since the future is uncertain, it is a waste to think about it”. 1 = completely disagree, 2 = disagree, 3 = neither agree nor disagree, 4 = agree, 5 = completely agree

* Indicates data from the 2020 wave.

**Table 2:** Descriptive statistics.

Variables	Mean	Std. Dev.	Min	Max
Dependent variable				
Loneliness	0.6513	0.4766	0	1
Explanatory variables				
Smartphone use	121.2319	132.3550	0	1380
Male	0.6970	0.4597	0	1
Age	53.8266	12.7165	22	87
Spouse	0.6707	0.4700	0	1
Children	0.5916	0.4916	0	1
Living alone	0.2023	0.4018	0	1
Living in rural areas	0.5726	0.4948	0	1
Education	15.0177	2.0961	9	21
Full-time employment	0.6327	0.4822	0	1
Household income	6,511,217	4,262,293	500,000	21,000,000
Log of household income	15.4432	0.7806	13.12	16.86
Household asset	24,100,000	31,900,000	1,250,000	125,000,000
Log of household asset	16.0954	1.4524	14.04	18.64
Financial literacy	0.7099	0.3305	0	1
Subjective health status	3.2738	1.1310	1	5
Future anxiety	3.7810	1.1488	1	5
Financial satisfaction	2.8510	1.0959	1	5
Depression	2.8871	1.2445	1	5
Myopic view of the future	2.6882	1.0048	1	5
Observations	2630			

**Table 3:** Distribution of loneliness by gender and age group.

Loneliness	Male		Female		Total
	Younger Subsample (<65)	Older Subsample (≥65)	Younger Subsample (<65)	Older Subsample (≥65)	
0	423	236	192	66	917
	31.57%	47.87%	27.63%	64.71%	34.87%
1	917	257	503	36	1713
	68.43%	52.13%	72.37%	35.29%	65.13%
Total	1340	493	695	102	2630
	100%	100%	100%	100%	100%

**Table 4:** Logit regression results of loneliness in 2022.

Variables	Dependent Variable: Loneliness			
	Model 1	Model 2	Model 3	Model 4
Smartphone use	−0.00146 **	−0.00144 **	−0.00171 ***	−0.00183 ***
	(0.000616)	(0.000614)	(0.000586)	(0.000613)
Male	0.00308	−0.106	−0.0233	0.00218
	(0.189)	(0.211)	(0.210)	(0.224)
Age	−0.0351 ***	−0.0338 ***	−0.0358 ***	−0.0366 ***
	(0.00806)	(0.00890)	(0.00859)	(0.00935)
Spouse	−0.0478	0.0192	0.0115	0.0132
	(0.197)	(0.202)	(0.190)	(0.194)
Children	−0.346 **	−0.315 **	−0.177	−0.180
	(0.146)	(0.150)	(0.142)	(0.146)
Living alone	−0.0777	−0.276	−0.164	−0.206
	(0.298)	(0.314)	(0.330)	(0.337)
Living in rural areas	−0.0204	−0.0327	−0.0752	−0.0864
	(0.174)	(0.171)	(0.181)	(0.190)
Education	0.0477	0.0671	0.0715	0.0778
	(0.0491)	(0.0535)	(0.0511)	(0.0541)
Full-time employment		0.200	0.130	0.146
		(0.152)	(0.151)	(0.153)
Log of HH income		−0.308 ***	−0.244 **	−0.259 **
		(0.115)	(0.120)	(0.125)
Log of HH assets		−0.0557	0.0706	0.0597
		(0.0610)	(0.0669)	(0.0727)
Financial literacy		0.328	0.345	0.389 *
		(0.206)	(0.219)	(0.218)
Subjective health status			−0.325 ***	−0.296 ***
			(0.0789)	(0.0953)
Future anxiety			0.335 ***	0.285 ***
			(0.0841)	(0.0788)
Financial satisfaction			−0.143	−0.104
			(0.102)	(0.107)
Depression				0.203 **
				(0.0962)
Myopic view of the future				0.0342
				(0.0592)
Constant	1.962 **	6.963 ***	4.219 **	3.821
	(0.932)	(1.724)	(2.042)	(2.379)
Observations	2630	2630	2630	2630
Log pseudolikelihood	−61,555,231	−60,957,684	−57,434,672	−56,874,487
Chi^2^ statistics	63.52	70.55	171.5	226.7
*p*-value	0.0000	0.0000	0.0000	0.0000

Robust standard errors in parentheses *** *p* < 0.01, ** *p* < 0.05, * *p* < 0.1.

**Table 5:** Logit regression results of loneliness in 2022 (subsample analysis by gender and age group).

Variables	Dependent Variable: Loneliness			
	Younger Subsample (<65)		Older Subsample (≥65)	
	Male	Female	Male	Female
Smartphone use	−0.000770	−0.00245 **	−0.000759	0.00200
	(0.000584)	(0.000961)	(0.000972)	(0.00359)
Age	0.0243 *	−0.0310 ***	−0.102 ***	−0.186 *
	(0.0139)	(0.0116)	(0.0307)	(0.112)
Spouse	−0.134	0.0565	0.347	0.445
	(0.272)	(0.322)	(0.582)	(0.719)
Children	−0.121	−0.181	−0.231	0.448
	(0.214)	(0.227)	(0.402)	(0.887)
Living alone	−0.354	−0.179	1.047	2.631 ***
	(0.339)	(0.401)	(0.649)	(0.956)
Living in rural areas	0.126	0.0341	0.0295	−0.212
	(0.231)	(0.208)	(0.265)	(0.516)
Education	0.0621	0.00174	−0.0837	0.244 *
	(0.0658)	(0.0707)	(0.0686)	(0.140)
Full-time employment	−0.362	0.328	−0.174	−1.058
	(0.288)	(0.263)	(0.292)	(1.086)
Log of HH income	−0.218	−0.0652	−0.338	−0.185
	(0.146)	(0.210)	(0.267)	(0.511)
Log of HH assets	0.107	0.0331	0.327 **	0.114
	(0.0709)	(0.0891)	(0.133)	(0.414)
Financial literacy	0.602 *	−0.0475	−0.202	2.468 *
	(0.313)	(0.304)	(0.503)	(1.283)
Subjective health status	−0.0213	−0.388 ***	−0.144	−0.279
	(0.0922)	(0.106)	(0.130)	(0.227)
Future anxiety	0.0591	0.325 ***	0.375 ***	0.786 **
	(0.0919)	(0.103)	(0.140)	(0.333)
Financial satisfaction	−0.136	−0.133	−0.219	0.0907
	(0.118)	(0.112)	(0.161)	(0.341)
Depression	0.563 ***	0.236 ***	0.350 ***	−0.0207
	(0.109)	(0.0884)	(0.125)	(0.283)
Myopic view of the future	0.159 **	0.0444	0.235	−0.462
	(0.0785)	(0.0993)	(0.146)	(0.355)
Constant	−1.350	2.730	6.847	6.149
	(2.440)	(2.852)	(4.205)	(8.177)
Observations	1340	695	493	102
Log pseudolikelihood	−18,776,852	−16,861,409	−8,716,859	−6,121,920
Chi^2^ statistics	102.4	70.84	56.59	26.32
*p*-value	0.0000	0.0000	0.0000	0.0497

Robust standard errors in parentheses *** *p* < 0.01, ** *p* < 0.05, * *p* < 0.1.

## Data Availability

Data is available upon request.

## Appendix A

**Table A1:** Proportion of male and female across different age groups in the 2020 population census.

Age Groups	Male (%)	Female (%)
20–24	6.16	5.60
25–29	6.23	5.67
30–34	6.48	5.90
35–39	7.38	6.76
40–44	8.28	7.58
45–49	9.93	9.12
50–54	9.47	8.78
55–59	7.89	7.45
60–64	7.41	7.16
65–69	7.76	7.77
70–74	9.30	9.86
75–79	6.12	7.21
80–84	4.74	6.32
85–89	2.84	4.82

Source: Statistics of Japan [35].
